# Transcriptome Profiling Reveals the Negative Regulation of Multiple Plant Hormone Signaling Pathways Elicited by Overexpression of C-Repeat Binding Factors

**DOI:** 10.3389/fpls.2017.01647

**Published:** 2017-09-21

**Authors:** Aixin Li, Mingqi Zhou, Donghui Wei, Hu Chen, Chenjiang You, Juan Lin

**Affiliations:** State Key Laboratory of Genetic Engineering, Institute of Plant Biology, School of Life Sciences, Fudan University Shanghai, China

**Keywords:** *Arabidopsis thaliana*, auxin, CBF transcription factor, plant hormone signaling, RNA-Seq, transcriptome analysis

## Abstract

C-repeat binding factors (CBF) are a subfamily of AP2 transcription factors that play critical roles in the regulation of plant cold tolerance and growth in low temperature. In the present work, we sought to perform a detailed investigation into global transcriptional regulation of plant hormone signaling associated genes in transgenic plants engineered with *CBF* genes. RNA samples from *Arabidopsis thaliana* plants overexpressing two *CBF* genes, *CBF2* and *CBF3*, were subjected to Illumina HiSeq 2000 RNA sequencing (RNA-Seq). Our results showed that more than half of the hormone associated genes that were differentially expressed in *CBF2* or *CBF3* transgenic plants were related to auxin signal transduction and metabolism. Most of these alterations in gene expression could lead to repression of auxin signaling. Accordingly, the IAA content was significantly decreased in young tissues of plants overexpressing *CBF2* and *CBF3* compared with wild type. In addition, genes associated with the biosynthesis of Jasmonate (JA) and Salicylic acid (SA), as well as the signal sensing of Brassinolide (BR) and SA, were down-regulated, while genes associated with Gibberellin (GA) deactivation were up-regulated. In general, overexpression of *CBF2* and *CBF3* negatively affects multiple plant hormone signaling pathways in *Arabidopsis*. The transcriptome analysis using *CBF2* and *CBF3* transgenic plants provides novel and integrated insights into the interaction between CBFs and plant hormones, particularly the modulation of auxin signaling, which may contribute to the improvement of crop yields under abiotic stress via molecular engineering using *CBF* genes.

## Introduction

Environmental stresses such as low or high temperatures not only restrain the growth and yield of agriculturally important crops but also limit their temporal and spatial distribution (Kasuga et al., 1999). Many plants possess the ability to survive freezing temperatures, which depends to a large extent on their capacity of cold acclimation (Thomashow, 2010). In this process, C-repeat binding factors (CBFs) are a group of key regulatory proteins that can activate downstream regulon to enhance cold resistance via binding to drought responsive element (DRE)/c-repeat transcription (CRT) elements (Zhou et al., 2011). CBFs belong to AP2 transcription factor family and six *CBF* genes have been identified in *Arabidopsis thaliana*, including *CBF1*, *CBF2*, *CBF3*, *CBF4, DDF1* and *DDF2* (also known as *DREB1b*, *DREB1c*, *DREB1a*, *DREB1d*, *DREB1f*, and *DREB1e*) (Sakuma et al., 2002; Haake et al., 2002; Magome et al., 2004). Among which *CBF1*, *CBF2*, and *CBF3* specifically play central roles in cold acclimation (Shinwari et al., 1998; Hannah et al., 2006; Maruyama et al., 2012; Kang et al., 2013). The expression of *CBF1*, *-2*, *-3* is induced by low temperature in a fast and transient manner in various plant species (Jaglo-Ottosen et al., 1998; Hsieh et al., 2002; Savitch et al., 2005; Benedict et al., 2006; Akhtar et al., 2012). In addition to increased freezing tolerance, overexpression of *CBF* genes also cause multiple morphological and biochemical changes, including dehydration and high salt stress tolerances (Kasuga et al., 1999), growth retardation, delayed flowering and reduced yields (Liu et al., 1998; Gilmour et al., 2000; Hsieh et al., 2002; Lee et al., 2004; Kasuga et al., 2004; Pino et al., 2008; Tong et al., 2009; Bhatnagar-Mathur et al., 2014), delayed leaf senescence and extended plant longevity (Sharabi-Schwager et al., 2010a,b), and a slight bluish-green tint of leaves (Gilmour et al., 2000). In summary, *CBF* overexpression widely affects plant physiological and developmental processes involving a complex transcriptional regulation system (Kendall et al., 2011; Park et al., 2015).

Plant hormones are key signal molecules regulating plant growth, development and stress tolerance (Peleg and Blumwald, 2011; Kurepin et al., 2013; Colebrook et al., 2014; Shi et al., 2015). Understanding the correlation between CBF-mediated biochemical changes and plant hormone signaling pathways will be an effective way to improve the plant adaption to environments through genetic engineering. Gibberellins (GAs) are the first class of plant hormones revealed that it can be regulated by *CBFs* (Hedden and Phillips, 2000; Shan et al., 2007). It has been reported that the dwarfism of *CBF1-ox* and *CBF3-ox* plants can be rescued by exogenous GA treatments (Achard et al., 2008; Zhou et al., 2017). CBF3 transcription factor targets the GA deactivation gene *GA2ox7* to reduce endogenous GA levels and caused the accumulation of DELLA proteins, leading to limited plant growth (Zhou et al., 2017). In other plant species such as tobacco, bioactive GA levels were also reduced by *CbCBF* from Capsella (Zhou et al., 2014). Some recent reports showed that *CBF2* overexpression might be involved in leaf response to ethylene via ABA signaling (Sharabi-Schwager et al., 2010a) and enhanced abiotic stress tolerance via cytokinin signaling (Huang et al., 2009). Besides, the expression of *CBFs* is negatively regulated by ethylene (Shi et al., 2012) and SA (Miura and Ohta, 2010), positively regulated by JA (Hu et al., 2013), and can be induced by exogenous ABA (Knight et al., 2004; Yang et al., 2011; Knight and Knight, 2012). These indicate that *CBFs* interact with different plant hormones, nevertheless little is known about detailed regulatory modes of hormone signaling genes in *CBF* overexpression plants. The modulation of more hormones such as auxins by *CBFs* also needs to be clarified.

Recently, two groups generated *cbfs* double and triple mutants using CRISPR/Cas9 system to reveal that three *CBF* genes play redundant and crucial roles in cold acclimation (Jia et al., 2016; Zhao et al., 2016). The genetic analysis clearly demonstrated that *CBF2* is more important than *CBF1* and *CBF3* in cold acclimation-mediated freezing resistance (Zhao et al., 2016). For further application of *CBF* genes in molecular breeding using transgenic technology, detailed investigation into global changes of gene expression pattern elicited by overexpression of *CBF* genes is required. Here, we used RNA-seq tools to analyze the transcriptome of *CBF2-ox* and *CBF3-ox Arabidopsis* plants. The transcriptome profiling is an effective and widely used method to investigate gene expression dynamics in response to abiotic stresses at a global level in many species (Zhu et al., 2013). We explored differentially expressed genes related to plant hormones with emphasis on auxin signaling. The negative regulation of multiple plant hormone signaling pathways in *CBF* overexpression plants were discussed.

## Materials and Methods

### Plant Materials

The wild type (WT) *Arabidopsis thaliana* (L.) Heynh. ecotype Wassileskija (*Ws*-2) and transgenic plants constitutively expressing *CBF2* (*CBF2-o*x) or *CBF3* (*CBF3-ox*) in this work were previously described (Gilmour et al., 2004). These plants were grown in Metro-Mix 200 soil under cycles of 16-h-light/8-h-dark in a growth chamber at constant 22°C. Two-week-old seedlings were used for detection of relative expression levels of selected genes.

### RNA Extraction, cDNA Library Construction and Illumina Sequencing

Total RNA was extracted from collected 2-week-old seedlings using ZR Plant RNA MiniPrep^TM^ Kit (ZYMO Research Corp., United States) and purified using RNAeasy Plant Mini Kit (Qiagen, Valencia, CA, United States) according to the manufacturer’s protocol. The quality and quantity of total RNA were examined by a 2100 Bioanalyzer (Aligent Technologies, Santa Clara, CA, United States) according to the manufacturer’s instruction. After purification, three biological replicates of RNA samples for each genotype were pooled and digested into 200-nt fragments (Agilent Technologies). The first strand cDNA was generated from RNA fragments by reverse transcriptase using random primers (Invitrogen, San Diego, CA, United States). Second-strand cDNA was synthesized using reaction buffer, dNTPs, RNase H and DNA polymerase I (Amersham Biosciences, Piscataway, NJ, United States). After second strand cDNA synthesis and adaptor ligation, 200-bp cDNA fragments were purified by gel electrophoresis and enriched by 18 cycles of PCR to construct the final sequencing cDNA libraries. Qualities of these libraries were evaluated by an Agilent Bioanalyzer and the ABI Step One Plus Real-Time PCR system (Applied Biosystems, Foster City, CA, United States). The constructed libraries were sequenced using the HiSeqTM 2000 Sequencing System (Illumina, San Diego, CA, United States) with single-end technology in a single run at Shanghai Genergy Biotechnology (Shanghai) Co., Ltd. (Shanghai, China) and subjected to 100 cycles of paired-end (2 × 100 bp) sequencing. RNA-seq data are deposited in the NCBI Sequencing Read Archive database and the accession numbers are SRR5665776, SRR5665777, and SRR5665778.

### Identification of Differentially Expressed Genes (DEGs)

Raw reads generated by high-throughput sequencing of *Ws*-2, *CBF2-ox* and *CBF3-ox* samples were cleaned by removing adaptor sequences, empty reads, and low-quality sequences (Ns > 5) (Cock et al., 2010). The sequence reads then were mapped to the *Arabidopsis* genome downloaded from TAIR10^1^ by TopHat2 (Kim D. et al., 2013)^2^ using the modified parameters of ‘no mismatch’ and ‘single copy.’ The expression level of each gene was normalized by calculating the Reads Per Kilobase per Million (RPKM) method using Cufflinks (Roberts et al., 2011) with default parameters. The raw read counts for each gene was calculated using HTSEQ v.0.6.0 (Anders et al., 2015). The Differentially expressed genes (DEGs) data were generated with DEGseq2 (Love et al., 2014). DEGs were identified with the criteria of Log_2_ Fold-Change (Log_2_FC) ≥ 1 and False Discovery Rate (FDR, Benjamini–Hochberg adjusted *P*-value) ≤ 0.01.

### Functional Annotation and Pathway Analysis

GO functional classifications and enrichment analysis for all DEGs were carried out by agriGO^3^ (Du et al., 2010). Singular Enrichment Analysis (SEA) tool was used in agriGO to produce pathway signaling. The hypergeometric method of statistical test was used and significance level was 0.05. All parameters were used default settings. The DEGs involve in plant hormone signaling pathways was selected using plant hormone signal transduction map in the database of Kyoto Encyclopedia of Genes and Genomes (KEGG)^4^.

### Relative mRNA Expression Level Analysis

Real-time quantitative reverse transcription-PCR (qRT-PCR) was applied to investigate gene expression patterns. The mRNA levels were measured using *SYBR Premix ExTaq^TM^ II* (Perfect Real Time) (TaKaRa, Dalian, China) according to manufacturer’s instructions. qRT-PCR was conducted using ABI 7500 (Applied Biosystems, Foster City, CA, United States). The reactions were performed using biological replicates different from RNA-seq samples in three independent experiments, and three technical replicates were used for each run. Values “Ct” obtained for all genes were normalized to that of an internal control *EF1α* gene from *Arabidopsis*. The relative expression level of each gene was calculated using the 2^-ΔΔC_T_^ method (Livak and Schmittgen, 2001). Statistical significances of differences between the sample and control plants were determined by Student’s test. The specific primers were designed using Primer Express software (Applied Biosystems, Foster City, CA, United States) and sequences were listed in Supplementary Table S1.

### Measurements of Endogenous Auxin (IAA) Contents of *Arabidopsis* by Enzyme-Linked Immunosorbent Assay (ELISA)

The endogenous auxin levels of *Arabidopsis* were estimated on three different types of tissues including young seedlings (S), leaves (L) and roots (R) of 2-week-old plants. Tissues of S, L and R from three plants were combined as one sample and three biological replicates were used for statistical tests. The auxin (IAA) contents were determined by enzyme linked immunosorbent assay (ELISA) as described previously (Chen and Zhao, 2008).

## Results

### Identification of *CBF-ox* Regulated Genes

**FIGURE 1:**
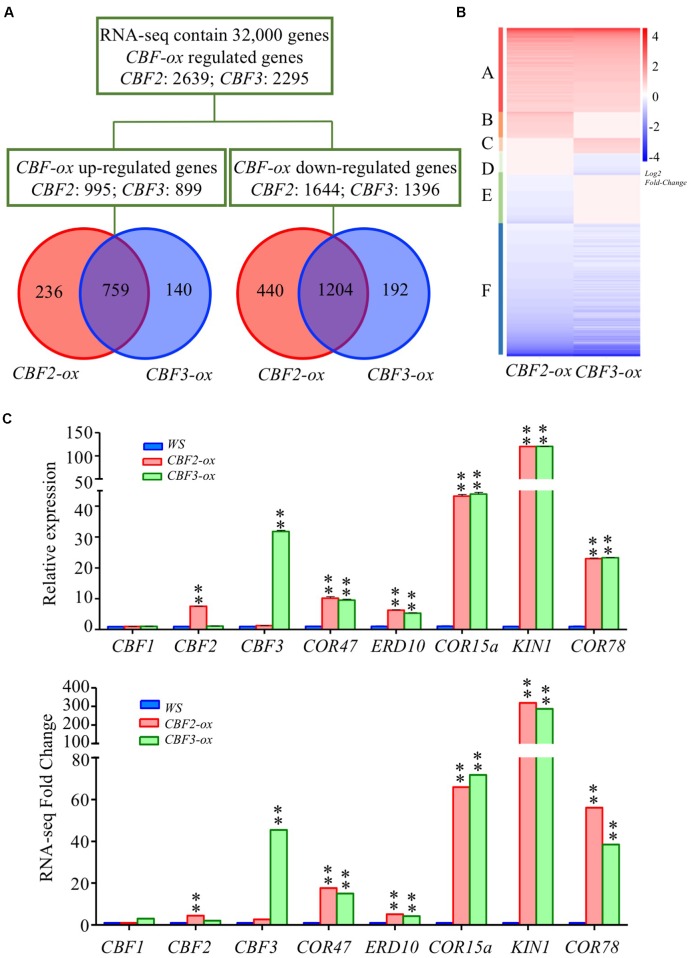
Overview of transcriptomes in *Ws*, *CBF2-ox* and *CBF3-ox* plants. **(A)** Venn diagram representing the total number of differentially expressed genes (DEGs) between *CBF2-ox* or *CBF3-ox* and *Ws*. *CBF-ox* up-regulated (log_2_ ≥ 1, FDR ≤ 0.01) or down-regulated (log_2_ ≤ -1, FDR ≤ 0.01) genes were shown. **(B)** Hierarchical cluster analysis of the DEGs. The heat-map was drawn with DEGseq of R package. **(C)** Verification of CBF regulon expression in *Ws*, *CBF2-ox* and *CBF3-ox* plants. Eight genes were selected for comparison of RNA-seq and qRT-PCR results. For RNA-seq data, ^∗∗^ represents FDR ≤ 0.01. The data of qRT-PCR were the means of three technical replicates ± SD (^∗∗^*P* ≤ 0.01; Student’s *t*-test). Three independent experiments were carried out with similar results.

A total number of 2639 genes (995 were up-regulated and 1644 were down-regulated) and 2295 genes (899 were up-regulated and 1396 were down-regulated) were found to be differentially expressed in *CBF2-ox* and *CBF3-ox* plants compared to WT (*Ws*-2) with at least two-fold differences (FDR ≤ 0.01), respectively. Venn diagrams were generated to show an overview of the DEGs (**Figure 1A**). The numbers of down-regulated genes were larger than up-regulated genes in both *CBF2-ox* and *CBF3-ox* plants. In line with previous analysis (Park et al., 2015), a large portion of DEGs (more than 70% for *CBF2-ox* and more than 80% for *CBF3-ox*) were coordinately changed in two transgenic lines. According to the expression patterns of DEGs in *CBF2-ox* and *CBF3-ox* plants, we generally divided genes regulated by *CBF2-ox* or *CBF3-ox* into six groups (**Figure 1B**). The DEGs specially regulated by *CBF2* were around two times as many as DEGs specially regulated by *CBF3*, indicating that *CBF2* may control bigger proportion of downstream CBF regulon than *CBF3*, which is in line with the conclusion generated from cold tolerance tests using double and triple *cbfs* mutants (Zhao et al., 2016). For validation of the transcriptome data, we selected three *CBF* genes and five CBF regulon for qRT-PCR examination in different samples (**Figure 1C**). The expression changes of these DEGs showed a good agreement between qRT-PCR and RNA-seq data.

### Functional Enrichment Analysis of *CBF-ox* Regulated Genes

**FIGURE 2:**
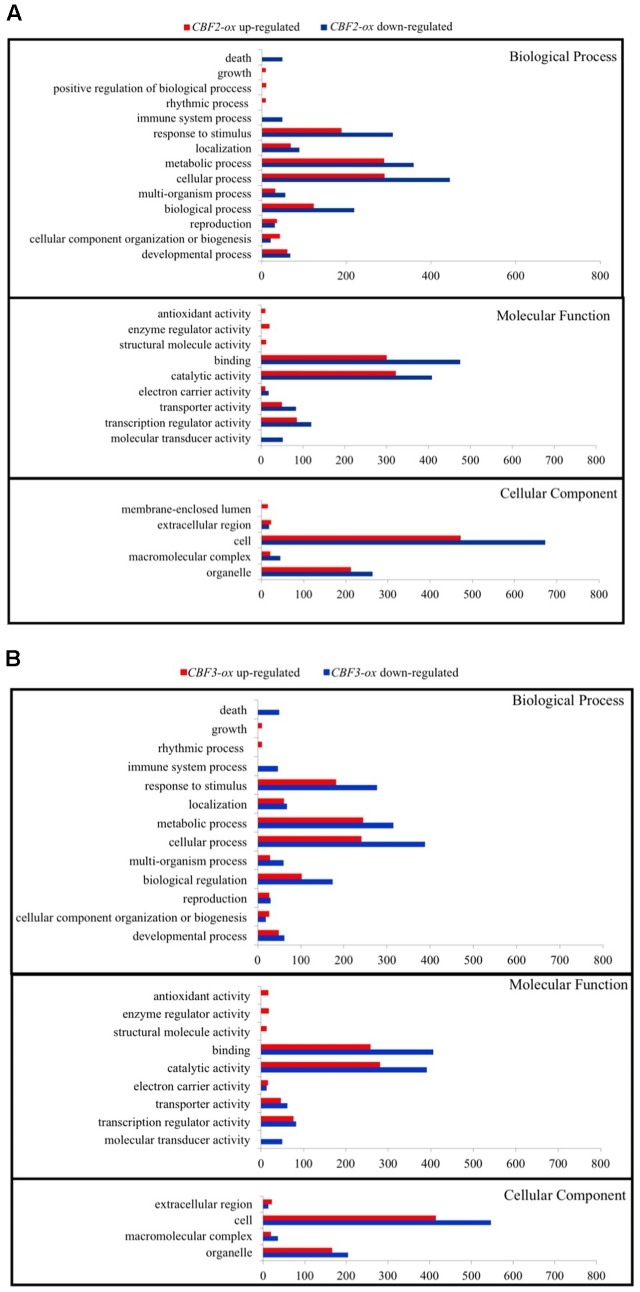
Signaling pathway enrichment of differentially expressed genes (DEGs) in *CBF2-ox* and *CBF3-ox Arabidopsis* plants. **(A)** GO functional classification of DEGs in *CBF2-ox* transcriptome; **(B)** GO functional classification of DEGs in *CBF3-ox* transcriptome.

**FIGURE 3:**
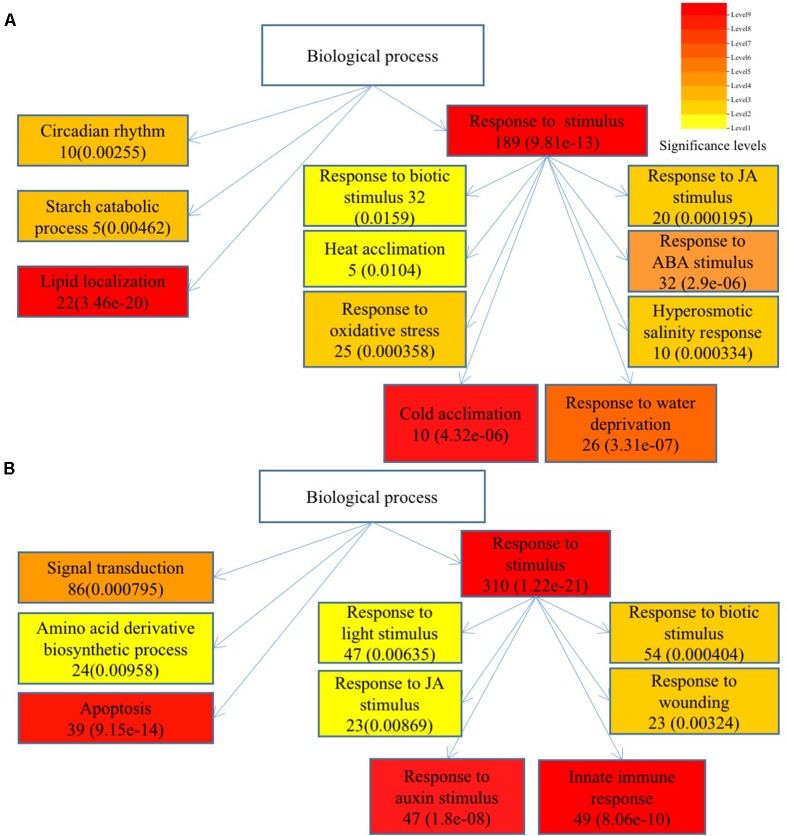
Significantly enriched biological process of differentially expressed genes in *CBF2-ox Arabidopsis* plants. **(A)** Enriched biological process of up-regulated genes. **(B)** Enriched biological process of down-regulated genes. Gene numbers are shown in the boxes and *e*-values are shown in parenthesis.

**FIGURE 4:**
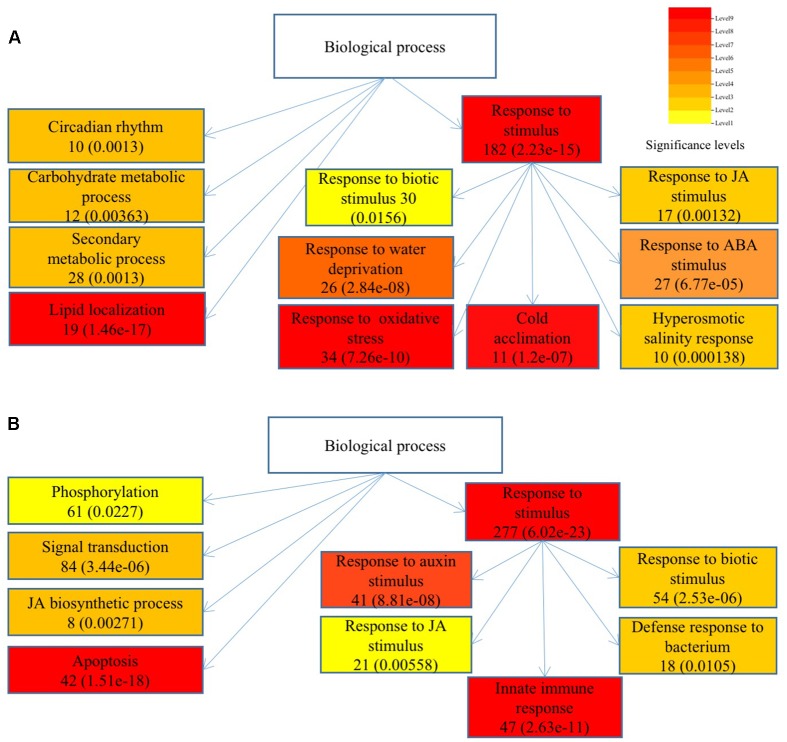
Significantly enriched biological process of differentially expressed genes in *CBF3-ox Arabidopsis* plants. **(A)** Enriched biological process of up-regulated genes. **(B)** Enriched biological process of down-regulated genes. Gene numbers are shown in the boxes and *e*-values are shown in parenthesis.

To understand the functions of DEGs and elucidate the metabolic or signal transduction pathways formed by these genes, we used SEA tool in agriGO^5^. All of the DEGs between *Ws*-2 and *CBF2-ox* or *CBF3-ox* plants were assigned to functional categories by GO analysis. DEGs were categorized into biological process, cellular component, and molecular functions, which included 14, 5, and 9 functional groups in *CBF2-ox* and 13, 4, and 9 functional groups in *CBF3-ox*, respectively (**Figure 2**). In *CBF2-ox* plants, the up-regulated genes were significantly enriched in GO terms of response to oxidative stress, lipid localization, cold acclimation, water deprivation, salinity response, JA response, and ABA response. The down-regulated genes were significantly enriched in GO terms of apoptosis, auxin response, and innate immune response (**Figure 3**). Enriched signaling pathways of up and down regulated genes of *CBF3-ox* showed high similarities with *CBF2-ox* (**Figure 4**). Notably, phosphorylation and secondary metabolic process were only detected in *CBF3-ox* while heat acclimation, amino acid derivative biosynthetic process and response to light stimulus were only detected in *CBF2-ox* plants.

### *CBF-ox* Influences Plant Hormone Related Genes

**FIGURE 5:**
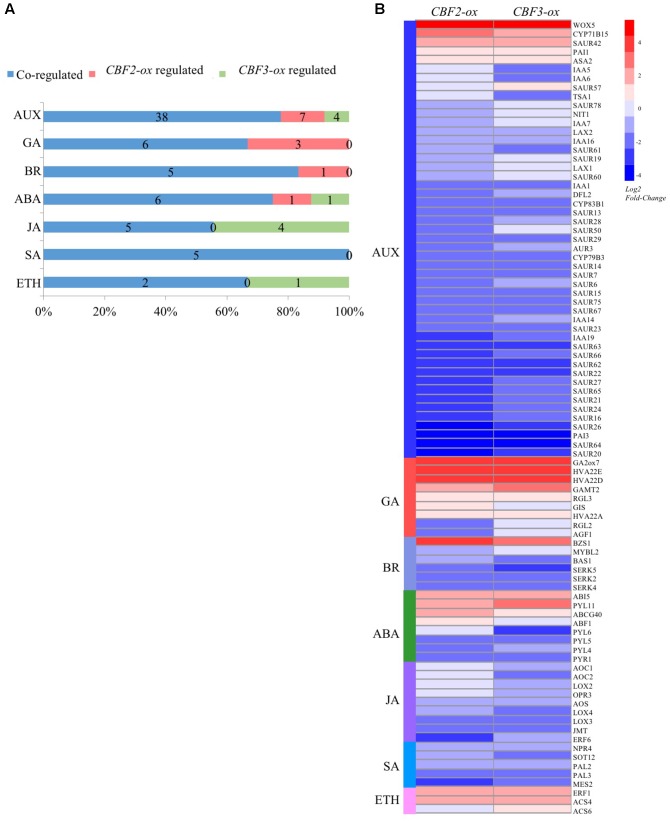
*CBF2-ox* and *CBF3-ox* regulate genes that are associated with auxin (AUX), gibberellin (GA), Brassinolide (BR), abscissic acid (ABA), Jasmonate (JA), Salicylic acid (SA) and ethylene (ETH) signaling. **(A)** The number of hormone associated genes regulated or co-regulated by *CBF2-ox* and *CBF3-ox*. **(B)** Heat-map showing expression fold change of 89 genes associated with plant hormones.

We identified 89 DEGs that participate in plant hormone modulation (**Figure 5A**). All these 89 genes were reported to play direct or indirect roles in hormone biosynthesis, molecular modification, transportation or degradation. Numerous elements containing “CCGAC,” the core sequence of CRT/DRE motif, were identified in the 3000 bp area upstream from coding regions of these genes (Supplementary Table S2). **Figure 5B** shows the expression patterns of the 89 genes in different plants. Among them, 17 DEGs were co-up-regulated by *CBF2* and *CBF3*, 5 of which were related to auxin (*WOX5*, *CYP71B15*, *ASA2*, *PAI1*, *SAUR42*) and 6 (*RGL3*, *GA2ox7*, *GAMT2*, *HVA22*, *HVA22A* and *HVA22D*) were related to GA. There are 2 DEGs were up-regulated by *CBF2-ox* only and 2 were *CBF3-ox* only, respectively. For down-regulation, 10 and 8 DEGs were *CBF2-ox* only and *CBF3-ox* only, respectively. Most of which were auxin or JA related genes. Besides, 49 DEGs were co-down-regulated and 32 of which were auxin related genes, indicating that *CBFs* widely affect plant hormone signaling especially toward auxins.

### *CBF-ox* Shows a Negative Regulation in Auxin Metabolism and Signaling

Auxins are the first class of defined plant hormones, which extensively control cell elongation, division and differentiation (Mano and Nemoto, 2012). In *CBF2-ox* and *CBF3-ox* plants, 49 DEGs were associated with auxin, which was more than any other plant hormones. Among these 49 auxin related genes, 5 genes were co-up-regulated and 32 genes were co-down-regulated by *CBF2-ox* and *CBF3-ox*. In addition, 7 genes were down-regulated by *CBF2-ox* only, 4 genes were down-regulated by *CBF3-ox* only and 1 gene was up-regulated by CBF3 only (**Figure 5A**). These indicated that *CBFs* generally showed a negative regulation in auxin associated genes.

**FIGURE 6:**
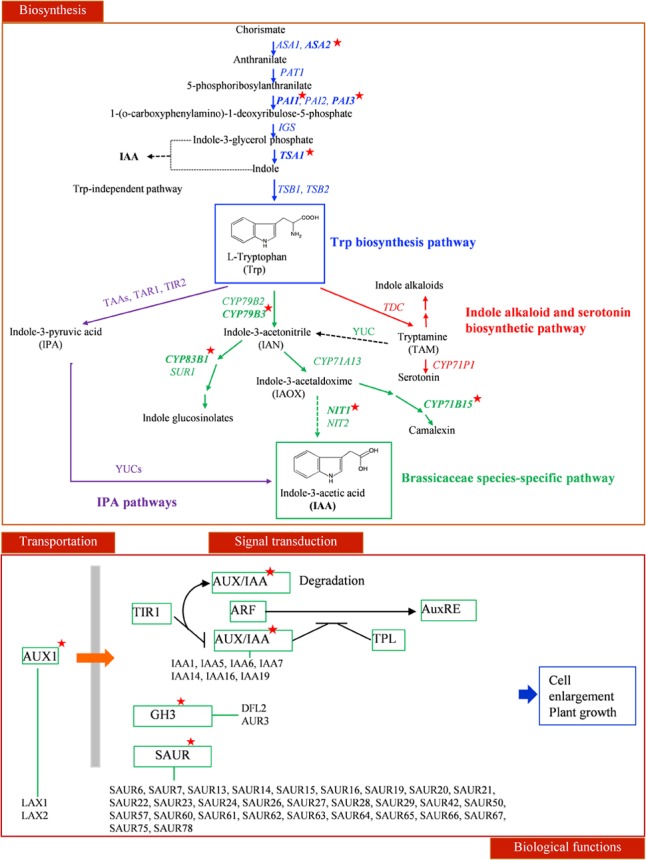
The influence of *CBF-ox* on auxin signaling pathway. For presumptive IAA biosynthesis pathways in plants, blue arrows indicate the tryptophan synthetic pathway in the chloroplast; a thin dashed black arrow denotes the tryptophan-independent IAA biosynthetic pathway; purple arrows indicate IPA biosynthetic pathway; red arrows indicate the indole alkaloid and serotonin biosynthetic pathway; green arrows indicate the Brassicaceae species-specific pathway. IAA transportation and signal transduction routes are also shown. Genes significantly changed in *CBF2-ox* or *CBF3-ox* plants are labeled by red stars.

**FIGURE 7:**
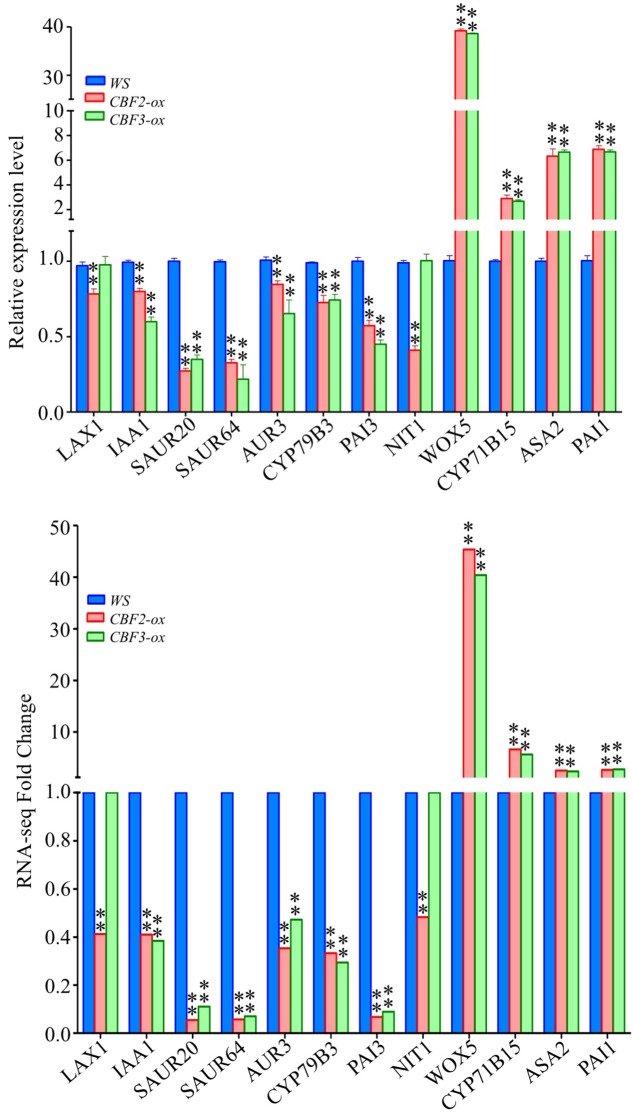
Expression of auxin pathway related genes in *WS*, *CBF2-ox* and *CBF3-ox* plants. Twenty genes were selected from the 49 auxin pathway genes for comparison of RNA-seq and qRT-PCR. For RNA-seq data, ^∗∗^ represents FDR ≤ 0.01. The data of qRT-PCR were the means of three technical replicates ± SD (^∗∗^*P* ≤ 0.01; Student’s *t*-test). Three independent experiments were carried out with similar results.

Auxin biosynthesis can be grouped as tryptophan dependent and independent pathways (Hull et al., 2000; Tromas and Perrot-Rechenmann, 2010). Two genes down-regulated by *CBF2-ox* and *CBF3-ox*, *CYP79B3* and *CYP83B1*, are critical enzymes in tryptophan dependent pathway (Mano and Nemoto, 2012). *NIT1* catalyzing the last step of IAA biosynthesis (Bartling et al., 1994; Lehmann et al., 2017) was also down-regulated by *CBF2-ox*. These suggested that *CYP79B3*, *CYP83B1* and *NIT1* are three potential CBF-regulated nodes in auxin biosynthesis. Meanwhile, auxin carrier *LAX1* and *LAX2* were also down-regulated. Multiple early auxin signaling transduction related genes such as small auxin up RNA genes (*SAURs*), *AUX/IAAs* and GH3 family genes were diversely changed (**Figure 6**). There were 2 up-regulated and 27 down-regulated *SAUR*s out of 59 *SAUR* genes in *Arabidopsis* (Li et al., 2015). The 7 down-regulated *IAA*s are auxin inducible and function as key regulators in auxin responses (Santner et al., 2009). The GH3-like gene *DFL2* is not induced by auxin but may play a role in hypocotyl elongation through light-controlled auxin modification (Takase et al., 2003). The repression of the other GH3-family gene *AHR3* could be due to a feedback of down-regulated auxin biosynthesis, for AHR3 protein conjugates free IAA to amino acids (Staswick et al., 2005). Twelve genes functioning in auxin metabolism and signaling transduction were subjected to qRT-PCR and their expression changes in *CBF2-ox* and *CBF3-ox* plants were in line with the RNA-seq data (**Figure 7**).

### *CBF-ox* Decreases Endogenous IAA Contents

**FIGURE 8:**
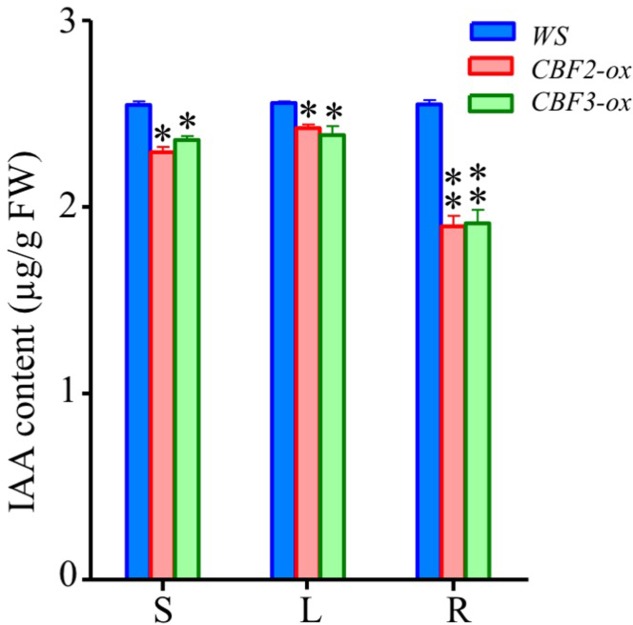
Auxin (IAA) contents in *WS*, *CBF2-ox* and *CBF3-ox* plants. S, the seedling of 2-week-old plants; L, leaves from 2-week-old plants; R, Roots of 2-week-old plants. The data are the means of three technical replicates ± SD (^∗^*P* ≤ 0.05, ^∗∗^*P* ≤ 0.01; Student’s *t*-test). Three independent experiments were carried out with similar results.

Since the auxin associated genes were largely repressed, we measured the contents of IAA, a major form of naturally occurring auxins (Mano and Nemoto, 2012), in *CBF2-ox* and *CBF3-ox* plants to verify the negative regulation of auxins level. The IAA contents in tissues of whole seedlings, leaves and roots of 2-week-old *Arabidopsis* were determined. Indeed, all three types of tissues of both *CBF2-ox* and *CBF3-ox* plants showed significantly decreased IAA levels (**Figure 8**). The difference of IAA levels between *CBF-ox* and *WT* in roots was bigger than whole seedlings and leaves, indicating that roots are most influenced by *CBF-ox* in auxin contents.

## Discussion

Ectopic expression of CBFs are useful tools for genetic engineering of stress tolerant plants (Zhou et al., 2011). The key point is how to eliminate the plant growth retardation caused by high expression of *CBF* genes. Previous studies surveyed around one-third of genes in *Arabidopsis* transcriptome and indicated that *CBF1-*, *CBF2-* and *CBF3-ox* shared similar affected gene sets (Fowler and Thomashow, 2002; Gilmour et al., 2004; Maruyama et al., 2004). In this study, we presented a genome-wide transcriptome analysis in *CBF2-ox* and *CBF3-ox* plants and identified 89 genes involved in plant hormone signaling and metabolism. Consistent with previous reports, DEGs regulated by *CBF2-ox* and *CBF3-ox* are largely overlapped and pathways enriched in GO analysis are quite similar, verifying the conclusion that *CBF2* and *CBF3* have redundant functions revealed by *cbfs* triple mutants (Jia et al., 2016). Meanwhile, *CBF2-ox* and *CBF3-ox* diversely regulate a series of genes. DEGs specifically regulated by *CBF2-ox* were mainly related to lipid localization, starch metabolic process, light stimulus response and regulation of transcription, while *CBF3-ox* specific DEGs were mainly related to oxidative stress response. These suggest that different strategies with respect to physiological and metabolic controls can be applied when using *CBF2* and *CBF3* to engineer crops.

It was shown that cold stress disturbed multiple auxin related genes (Lee et al., 2005). Those genes, such as auxin-inducible *SAUR* family genes, were also largely impacted in *CBF2-ox* and *CBF3-ox* plants (**Figure 5** and Supplementary Table S1). The fact that IAA levels were significantly reduced in *CBF2-ox* and *CBF3-ox* young tissues, especially in roots, is another agreement with previous study showing that *DR5* gene promoter activity, a reporter of endogenous auxin level, was downregulated in roots under cold treatments (Lee et al., 2005). The genes suppressed by *CBFs* are mainly related to the first and last step of IAA biosynthesis originated from tryptophan, as well as auxin transportation and signal transduction. Moreover, the gene with biggest changes in the list of 89 hormone related ones is WUSCHEL HOMEOBOX 5 (*WOX5*), a transcription factor playing a central role in stem cell maintenance in different meristem types (Pi et al., 2015). The up-regulation of *WOX5* suggests that CBF can inhibit the stem cell differentiation, which is consistent with the contribution of CBFs to delayed leaf senescence and extended longevity (Sharabi-Schwager et al., 2010a). Although it is not clear whether these genes are direct targets of CBF proteins yet, our transcriptome data provide a relatively comprehensive investigation of effects on auxin signaling from CBFs. The repression of auxin signaling can be another reason of plant growth retardation widely observed in *CBF-ox* plants in addition to influence on GA signaling (Achard et al., 2008; Zhou et al., 2017), and can also explain the reason why dwarf phenotype of *CBF*-ox *Arabidopsis* plants could only be partially rescued by GA application (Zhou et al., 2017).

Compared with old microarray data (Fowler and Thomashow, 2002), we detected more GA associated genes changed in *CBF2-ox* and *CBF3-ox* plants. Besides the reported GA2-oxidase gene *GA2ox7* and DELLA gene *RGL3* (Zhou et al., 2017), a GA methyl transferase gene *GAMT2* was significantly up-regulated in *CBF3-ox* plants. GAMT enzyme transfers active GA into inactive form (Varbanova et al., 2007; Doherty et al., 2009; Kim Y. et al., 2013) and can be a novel regulatory node of GA controlled by *CBF3*. Further, *HVA22*, *HVA22A* and *HVA22D* were up-regulated by both *CBF2-ox* and *CBF3-ox*. These three genes serve as repressors of programmed cell death mediated by GA (Guo and Ho, 2008). Surprisingly, another GA signaling repressor DELLA gene *RGL2* was down-regulated by *CBF2*, suggesting that *CBF2* may possess new functions that are different from *CBF1* and *CBF3* in GA regulation. For other hormones, although the regulation of *CBFs* and cold responses from ABA, JA and ethylene have been widely investigated, few information of CBF-caused genome wide influence in these hormones are reported. Here we show that *CBF2-ox* and *CBF3-ox* also impacted multiple aspects of plant hormones other than auxin and GA (**Figure 7**). The genes associated with biosynthesis (*PAL2* and *PAL3*) (Huang et al., 2010) and modification (*MES2* and *SOT12*) (Baek et al., 2010) of SA as well as the SA receptor (*NPR4*) (Fu et al., 2012) were coordinately repressed in *CBF2-ox* and *CBF3-ox* plants. Genes that are similarly down-regulated by CBF2 and CBF3 also include receptor kinase genes *SERK2, -4, -5* involved in BR signaling (Gou et al., 2012), key enzyme genes in JA biosynthesis such as *AOC1*, *-2*, *LOX2*, *-3*, *-4* and *OPR3* (Schaller et al., 2000), positive regulator gene *ERF6* in JA signaling and MeJA formation associated gene *JMT* (Seo et al., 2001). For ABA, receptor genes (Park et al., 2011) *PYL4*, *-5*, *-6* were down-regulated while *PYL11* was up-regulated. Regulator genes *ABF1* and *ABI5* as well as transportation gene *ABCG40* (Choi et al., 2000; Finkelstein et al., 2005) were all up-regulated. The ethylene biosynthesis gene *ACS4, ACS6* (Tsuchisaka et al., 2009) and positive regulator gene *ERF1* (Moffat et al., 2012) were up-regulated, which is in agreement with the study of positive correlation between ethylene biosynthesis and cold tolerance (Catalá et al., 2014). The recent transcriptome analyses performed in *cbfs* triple mutants also identified a series of hormone related genes (Jia et al., 2016; Zhao et al., 2016). Jia et al. reported 26 CBF-regulated genes associated with plant hormones and Zhao et al. showed 11 CBF-activated hormone related genes. Similar groups of genes were presented in our data, such as *GAMT2*, *RGL3* and *HVA* genes related to GA signaling, *BZS1* related to BR signaling, and *ABCG40* related to ABA signaling. Notably, a big proportion of hormone related genes detected in our work, including *GA2ox7*, *WOX5* and some of *SAUR* genes, are not detected in these two data sets of *cbfs* triple mutants, which can be due to different plant growth conditions and experimental procedures. More important, gene overexpression produced by transgenic technologies can trigger more extensive molecular changes and will help to identify pathway components that might remain undetected using loss-of-function analysis especially for functionally redundant genes (Prelich, 2012). Thus, our investigation would be a fine supplement to the research on CBF-dependent regulatory network and could guide the utilization of plant hormones in CBF transgenic plants.

**FIGURE 9:**
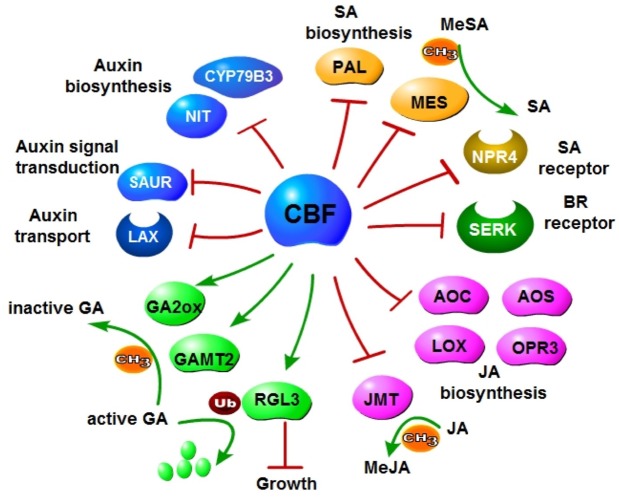
Negative regulation of multiple plant hormone pathways elicited by CBF overexpression. CBF2 and CBF3 repress biosynthesis of auxin, SA and JA; negatively regulate receptors of BR and SA; promote deactivation of GA.

In summary, *CBFs* negatively regulate auxin, GA, SA, JA and BR signaling, and disturb auxin, GA, SA and JA metabolism including biosynthesis, modification and transportation (**Figure 9**). All these hormones are associated with plant growth and development, implying a key role of *CBFs* in the balance of enhanced stress tolerance and restrained growth for plants to survive the environmental changes. Our data revealed more potential targets for crop improvement using genetic engineering approaches. The comprehensive modulation of *CBFs* and downstream regulons will hopefully realize the production of hardy crops without reduced growth.

## Author Contributions

JL, MZ, and AL were responsible for the overall design and conduct of experiments. MZ prepared plant tissues and performed RNA extraction and sequencing. AL and CY conducted the seq data analysis. DW and HC carried out the qRT-PCR and auxin content quantification. JL, AL, and MZ took the lead on manuscript development. All authors read and approved the final manuscript.

## Conflict of Interest Statement

The authors declare that the research was conducted in the absence of any commercial or financial relationships that could be construed as a potential conflict of interest.

## Footnotes

1 http://www.arabidopsis.org

2 http://tophat.cbcb.umd.edu

3 http://bioinfo.cau.edu.cn/agriGO/

4 http://www.kegg.jp/kegg-bin/show_pathway?map=ath04075&show_description=show

5 http://bioinfo.cau.edu.cn/agriGO/analysis.php
