# Supplementary material for: Transcriptome Profiling Reveals the Negative Regulation of Multiple Plant Hormone Signaling Pathways Elicited by Overexpression of C-Repeat Binding Factors
Source: Front Plant Sci. 2017 Sep 21;8:1647. doi: 10.3389/fpls.2017.01647 (PMC5613223; doi:10.3389/fpls.2017.01647)
Supplement: Supplementary file 1 [file Table_1.PDF]

Supporting Information

Table S1 Primers for qRT-PCR in this paper

Gene Name	Primer Name	Sequence (5’--3’)
CBF1	Real-CBF1F	GGAGACAATGTTTGGGATGC
	Real-CBF1R	CGACTATCGAATATTAGTAACTCC
CBF2	Real-CBF2F	CGACGGATGCTCATGGTCTT
	Real-CBF2R	TCTTCATCCATATAAAACGCATCTTG
CBF3	Real-CBF3F	TTCCGTCCGTACAGTGGAAT
	Real-CBF3R	AACTCCATAACGATACGTCGTC
COR47	AT1G20440-F	GTCCTTTCTTATCTTCCTCTCCTTC
	AT1G20440-R	AGAACAAGCCTAGTGTCATCG
ERD10	AT1G20450-F	CTTCTCACCGTCTTCACCTTC
	AT1G20450-R	AGAAAACAAGCCAAGTCTCCT
COR15a	AT2G42540-F	ATGCCTCTTTTGTTTTATCCGTC
	AT2G42540-R	AGTCGGCCAGAAAACTCAG
KIN1	AT5G15960-F	AGAATGCCTTCCAAGCCG
	AT5G15960-R	CATCCGATACACTCTTTCCCG
COR78	AT5G52310-F	TCCAAAGTTACTGATCCCACC
	AT5G52310-R	GTCTCTTTCAAATTGTCCTGGC
LAX1	AT5G01240-F	CCTCTTTTCTCCATGGTCAGG
	AT5G01240-R	CTTGGTTTCCACATCGCATG
LAX2	AT2G21050-F	TGACCACTTACACTGCTTGG
	AT2G21050-R	AATGCTTCACTCCTTCTACCTG
IAA1	AT4G14560-F	AGAGCTTCGTTTGGGATTACC
	AT4G14560-R	GGCCATCCAACGATTTGTG
SAUR20	AT5G18020-F	GATCGTTAAGCCACCCATTG
	AT5G18020-R	CTTGCAGTGTACGTAGGTGAG
SAUR64	AT1G29450-F	ACTCAGGTAACTTAGCGGAAAAG
	AT1G29450-R	CCTCCACAGGAAAAGGATCTC
AUR3	AT4G37390-F	TCACCGAGTTTCTCACAAGC
	AT4G37390-R	CTTTGCCTTTGTCTAATCCCG
CYP79B3	AT2G22330-F	GAGAAGGTAAGAGAACTCAGATCG
	AT2G22330-R	GCGTTTGATGGGTTGTCTG
NIT1	AT3G44310-F	CAACTGTCCAAAACGCAACTC
	AT3G44310-R	GCCTCCACAATATACTTCTCCG
WOX5	AT3G11260-F	GAATGTTTTCTATTGGTTTCAGAATCAT
	AT3G11260-R	ACCTTCTCTTCCTCTTGACAATC
CYP71B15	AT3G26830-F	AGAAGCAAGAGAACGATGGAG
	AT3G26830-R	GATCAGCTCGGTCATTCCC
ASA2	AT2G29690-F	CTTAGAGCTGTACTTCCCGTTG
	AT2G29690-R	CTCCAAAGCCTCCACTGTAAG
PAI1	AT1G07780-F	GCGAACCATTACCATGAAGC
	AT1G07780-R	GATGAGAACACGATACTGAGAGC
PAI3	AT1G29410-F	CCTTAGGAGCTACATTCTCTGTG
	AT1G29410-R	ATCTCCACCTCCATCCTAGAG
TSA1	AT1G52410-F	GGAAGAGATGGAACGTGAATTTG
	AT1G52410-R	TCCTAGTCCAAGAACCTCTGG
Actin2	R-actin2F	TGAGAGATTCAGATGCCCAGAA
	R-actin2R	TGGATTCCAGCAGCTTCCAT
