# Supplementary material for: Transcriptome Profiling Reveals the Negative Regulation of Multiple Plant Hormone Signaling Pathways Elicited by Overexpression of C-Repeat Binding Factors
Source: Front Plant Sci. 2017 Sep 21;8:1647. doi: 10.3389/fpls.2017.01647 (PMC5613223; doi:10.3389/fpls.2017.01647)
Supplement: Supplementary file 2 [file Table_2.PDF]

Supporting Information

Table S2 DEGs related to plant hormone

Gene locus	Name	CBF2FC[1]	CBF3FC	CCGAC[2]	Descriptions	References
Auxin						
AT5G01240	LAX1	-1.27603	/	4	auxin influx carriers	Robert et al., 2015
AT2G21050	LAX2	-1.18544	-1.06847	0	auxin influx carriers	
AT4G14560	IAA1	-1.28430	-1.37877	1	auxin-responsive protein 1	Zenser et al., 2001
AT1G15580	IAA5	/	-1.66209	3	early auxin-induced protein 5	
AT1G52830	IAA6	/	-2.20491	2	auxin-induced protein 6	
AT3G23050	IAA7	-1.09725	/	3	auxin-induced protein 7	
AT4G14550	IAA14	-2.19716	-1.02088	1	auxin-induced protein 14	
AT3G04730	IAA16	-1.21850	-1.04655	4	auxin-induced protein 16	
AT3G15540	IAA19	-2.29614	-2.21078	4	auxin-induced protein 19	
AT2G21210	SAUR6	-1.87953	-1.12013	1	small auxin-up RNA 6	Gil et al., 1994
AT2G21200	SAUR7	-1.79465	-1.33631	1	small auxin-up RNA 7	
AT4G38825	SAUR13	-1.36406	-1.52632	9	small auxin-up RNA 13	
AT4G38840	SAUR14	-1.70273	-1.51172	0	small auxin-up RNA 14	
AT4G38850	SAUR15	-1.94484	-1.80225	1	small auxin-up RNA 15	
AT4G38860	SAUR16	-3.04270	-1.87704	1	small auxin-up RNA 16	
AT5G18010	SAUR19	-1.24227	/	1	small auxin-up RNA 19	
AT5G18020	SAUR20	-4.19260	-3.17594	2	small auxin-up RNA 20	
AT5G18030	SAUR21	-2.90569	-2.01076	1	small auxin-up RNA 21	
AT5G18050	SAUR22	-2.67051	-2.68791	1	small auxin-up RNA 22	
AT5G18060	SAUR23	-2.24674	-1.84829	1	small auxin-up RNA 23	
AT5G18080	SAUR24	-2.93492	-2.09225	2	small auxin-up RNA 24	
AT3G03850	SAUR26	-3.77858	-2.52700	3	small auxin-up RNA 26	
AT3G03840	SAUR27	-2.80714	-2.23637	3	small auxin-up RNA 27	
AT3G03830	SAUR28	-1.38963	-1.25423	3	small auxin-up RNA 28	
AT3G03820	SAUR29	-1.49907	-1.89739	2	small auxin-up RNA 29	
AT2G28085	SAUR42	1.87238	1.82618	4	small auxin-up RNA 42	
AT4G34760	SAUR50	-1.42842	/	1	small auxin-up RNA 50	
AT3G53250	SAUR57	/	1.37339	0	small auxin-up RNA 57	
AT1G20470	SAUR60	-1.27759	/	1	small auxin-up RNA 60	
AT1G29420	SAUR61	-1.22760	-1.55971	1	small auxin-up RNA 61	
AT1G29430	SAUR62	-2.64959	-2.84371	1	small auxin-up RNA 62	
AT1G29440	SAUR63	-2.44732	-3.10358	1	small auxin-up RNA 63	
AT1G29450	SAUR64	-4.12067	-3.81451	0	small auxin-up RNA 64	
AT1G29460	SAUR65	-2.84889	-2.23952	1	small auxin-up RNA 65	
AT1G29500	SAUR66	-2.60435	-1.59698	0	small auxin-up RNA 66	
AT1G29510	SAUR67	-2.10273	-1.87036	1	small auxin-up RNA 67	
AT5G27780	SAUR75	-2.09262	-1.76196	2	small auxin-up RNA 75	
AT1G72430	SAUR78	-1.02037	/	0	small auxin-up RNA 78	Li et al., 2015
AT4G37390	AUR3	-1.50010	-1.08222	1	IAA-amido synthase	Mano and Nemoto, 2012
AT2G22330	CYP79B3	-1.58571	-1.76192	0	Trp-specific P450 monooxygenase	Hull et al., 2000
AT3G26830	CYP71B15	2.73081	2.49404	0	cytochrome P450 enzyme	Mano and Nemoto, K. 2012
AT2G29690	ASA2	1.33080	1.23692	0	anthranilate synthase	
AT4G31500	CYP83B1	-1.29429	-1.38247	0	cytochrome P450 monooxygenase	
AT1G07780	PAI1	1.40680	1.47054	3	phosphoribosylanthranilate isomerase 1	
AT1G29410	PAI3	-3.88011	-3.47406	1	phosphoribosylanthranilate isomerase 3	
AT1G52410	TSA1	/	-1.43774	0	Trp synthase α	
AT4G03400	DFL2	/	-1.18054	0	GH3-related gene	
AT3G44310	NIT1	-1.05044	/	1	nitrilase 1	Lehmann et al., 2017
AT3G11260	WOX5	5.50348	5.33624	2	Wuschel (WUS) family of homeodomain transcription factors	Gonzali et al., 2005
ETH						
AT2G22810	ACS4	2.17665	2.1474	4	aminocyclopropane-1-carboxylate synthase 4	Tsuchisaka et al., 2009
AT4G11280	ACS6	/	1.09526	0	aminocyclopropane-1-carboxylate synthase 6	
AT3G23240	ERF1	2.38444	2.27496	1	ERF (ethylene response factor) subfamily of AP2	Cheng et al., 2013
ABA						
AT2G38310	PYL4	-1.34197	-1.45439	2	PYR/PYL/RCAR family protein	Wang et al., 2013
AT5G05440	PYL5	-1.46018	-1.02995	2	PYR/PYL/RCAR family protein	
AT2G40330	PYL6	/	-2.35488	1	PYR/PYL/RCAR family protein	
AT5G45860	PYL11	2.23786	2.74767	3	PYR/PYL/RCAR family protein	
AT4G17870	PYR1	-1.65081	-1.41474	2	PYR/PYL/RCAR family protein	

AT1G49720	ABF1	1.22193	/	1	abscisic acid response elements-binding factor 1	Choi et al., 2000
AT2G36270	ABI5	2.58758	2.17374	2	basic leucine zipper transcription factor family	Finkelstein et al., 2005
AT1G15520	ABCG40	1.79623	1.56989	1	ABC transporter family	Kang et al., 2010
JA						
AT3G25760	AOC1	/	-1.10329	0	allene oxide cyclase	Wasternack et al., 2012
AT3G25770	AOC2	/	-1.32762	3	allene oxide cyclase	
AT5G42650	AOS	-1.07754	-1.21593	3	allene oxide synthase	
AT3G45140	LOX2	/	-1.25921	0	lipoxygenase	
AT1G17420	LOX3	-1.37719	-1.84022	2	lipoxygenase	
AT1G72520	LOX4	-1.22645	-1.46350	1	lipoxygenase	
AT1G19640	JMT	-2.03624	-2.19851	2	S-adenosyl-L-methionine: jasmonic acid carboxyl methyltransferase	Seo et al., 2001
AT2G06050	OPR3	/	-1.00021	0	12-oxophytodienoate reductase	Schaller et al., 2000
AT4G17490	ERF6	-2.26832	-1.03698	1	ERF (ethylene response factor) subfamily of AP2	Moffat et al., 2012
BR						
AT4G39070	BZS1	3.70887	3.54661	2	brassinosteroids-regulated BZR1 target (BRBT) gene	Fan et al., 2012
AT2G26710	BAS1	-1.26032	-1.28889	1	cytochrome p450 family	Turk et al., 2005
AT1G71030	MYBL2	-1.23667	/	2	myb family transcription factor	Ye et al., 2012
AT1G34210	SERK2	-1.93865	-1.6686	1	plasma membrane LRR receptor-like serine threonine kinase	Gou et al., 2012
AT2G13790	SERK4	-2.01036	-2.05516	0	plasma membrane LRR receptor-like serine threonine kinase	
AT2G13800	SERK5	-1.79155	-2.27216	1	plasma membrane LRR receptor-like serine threonine kinase	Wu et al., 2015
GA						
AT3G03450	RGL2	-1.33567	/	1	GRAS family of transcription factors	Colebrook et al., 2014
AT5G17490	RGL3	1.43505	1.57084	0	GRAS family of transcription factors	
AT1G50960	GA2ox7	4.25083	3.69787	2	gibberellin 2-oxidase	
AT5G56300	GAMT2	2.57540	2.74767	1	gibberellic acid methyltransferase 2	Varbanova et al., 2007
AT5G50720	HVA22	4.03485	4.02258	4	ABA/stress-induced protein	Guo and Ho, 2008
AT4G24960	HVA22D	3.70547	3.86619	4	ABA/stress-induced protein	
AT1G74520	HVA22A	1.33322	1.35799	2	ABA/stress-induced protein	
AT4G35390	AGF1	-1.41707	/	2	AT-hook motif nuclear localized protein 25	Matsushita et al., 2007
AT3G58070	GIS	1.36529	/	3	Putative transcription factor	An et al., 2012
SA						
At3g53260	PAL2	-1.26514	-1.03107	1	phenylalanine lyase	Dempsey et al., 2011
At5g04230	PAL3	-1.93569	-1.90886	2	phenylalanine ammonialyase (PAL) gene family	
At2g23600	MES2	-2.33546	-2.02076	2	methyl salicylate esterase	
At2g03760	SOT12	-1.08085	-1.36158	0	brassinosteroid sulfotransferase	
AT4G19660	NPR4	-1.00749	-1.00893	0	NPR1-LIKE PROTEIN 4	Fu et al., 2012

[1] FC (fold change): The relative expression value of genes in CBF2-ox or CBF3-ox versus in WT, the ratio was then converted to log2 value. Positive value means up regulate, negative value means down regulate. The larger the absolute value is the more significance the change is.

[2] Both strands of 3000bp upstream of gene coding area have been searched.

[3] '/' : no significant change

REFERENCES

An, L., Zhou, Z., Su, S., Yan, A., and Gan, Y. (2012). GLABROUS INFLORESCENCE STEMS (GIS) is required for trichome branching through gibberellic acid signaling in *Arabidopsis*. *Plant Cell Physiol*. 53, 457-469.

Cheng, M. C., Liao, P. M., Kuo, W. W., and Lin, T. P. (2013). The *Arabidopsis* ETHYLENE RESPONSE FACTOR1 regulates abiotic stress-responsive gene expression by binding to different cis-acting elements in response to different stress signals. *Plant Physiol.* 162,1566-1582.

Choi, H., Hong, J., Ha, J., Kang, J., and Kim, S.Y. (2000). ABFs, a family of ABA-responsive element binding factors. *J. Biol. Chem*. 275, 1723-1730.

Colebrook, E. H., Thomas, S. G., Phillips, A. L., and Hedden, P. (2014). The role of gibberellin signaling in plant responses to abiotic stress. *J. Exp. Biol*. 217, 67-75.

Dempsey, D. A., Vlot, A. C., Wildermuth, M. C., and Klessig, D. F. (2011). Salicylic acid biosynthesis and metabolism. *Arabidopsis Book.* 9, e0156.

Fan, X. Y., Sun, Y., Cao, D. M., Bai, M. Y., Luo, X. M., Yang, H. J., et al. (2012). BZS1, a B-box protein, promotes photomorphogenesis downstream of both brassinosteroid and light signaling pathways. *Mol Plant.* 5, 591-600.

Finkelstein, R., Gampala, S. S., Lynch, T. J., Thomas, T. L., and Rock, C. D. (2005). Redundant and distinct functions of the ABA response loci ABA-INSENSITIVE(ABI)5 and ABRE-BINDING FACTOR(ABF)3. *Plant Mol. Biol.* 59, 253-267.

Fu, Z. Q., Yan, S., Saleh, A., Wang, W., Ruble, J., Oka, N., et al. (2012). NPR3 and NPR4 are receptors for the immune signal salicylic acid in plants. *Nature*. 486, 228-232.

Gil, P., Liu, Y., Orbović, V., Verkamp, E., Poff, K. L., and Green, P. J. (1994). Characterization of the auxin-inducible SAUR-AC1 gene for use as a molecular genetic tool in Arabidopsis. *Plant Physiol*. 104,777-784.

Gonzali, S., Novi, G., Loreti, E., Paolicchi, F., Poggi, A., Alpi, A., et al. (2005). A turanose-insensitive mutant suggests a role for WOX5 in auxin homeostasis in *Arabidopsis thaliana*. *Plant J*. 44, 633-645.

Gou, X., Yin, H., He, K., Du, J., Yi, J., Xu, S., et al. (2012). Genetic evidence for an indispensable role of somatic embryogenesis receptor kinases in brassinosteroid signaling. *PLoS Genet*. 8, e1002452.

Guo, W.J., and Ho, T. H. (2008). An abscisic acid-induced protein, HVA22, inhibits gibberellin-mediated programmed cell death in cereal aleurone cells. *Plant Physiol.* 147, 1710-1722.

Hull, A. K., Vij, R., and Celenza, J. L. (2000). Arabidopsis cytochrome P450s that catalyze the first step of tryptophan-dependent indole-3-aceticacid biosynthesis. *Proc. Natl. Acad. Sci.* USA 97, 2379-2384.

Kang, J., Hwang, J. U., Lee, M. Kim, Y. Y., Assmann, S. M., Martinoia E, et al. (2010). PDR-type ABC transporter mediates cellular uptake of the phytohormone abscisic acid. *Proc. Natl. Acad. Sci.* USA 107, 2355-2360.

Lehmann, T., Janowitz, T., Sánchez-Parra, B., Alonso, M.P., Trompetter, I., Piotrowski, M., et al. (2017). Arabidopsis NITRILASE 1 contributes to the regulation of root growth and development through modulation of auxin biosynthesis in seedlings. *Front Plant Sci.*, 8:36. doi:10.3389/fpls.2017.00036.eCollection.

Li, Z. G., Chen, H. W., Li, Q. T., Tao, J. J., Bian, X. H., Ma, B., et al. (2015). Three SAUR proteins SAUR76, SAUR77 and SAUR78 promote plant growth in Arabidopsis. *Sci. Rep.* 5:12477.

Mano, Y., and Nemoto, K. (2012). The pathway of auxin biosynthesis in plants. *J. Exp. Bot.* 63:2853-2872. doi:10.1093/jxb/ers091.

Matsushita, A., Furumoto, T., Ishida, S., and Takahashi, Y. (2007). AGF1, an AT-hook protein, is necessary for the negative feedback of AtGA3ox1 encoding GA 3-oxidase. *Plant Physiol*. 143, 1152-1162.

Moffat, C. S., Ingle, R. A., Wathugala, D. L., Saunders, N. J., Knight, H., and Knight, M. R. (2012). ERF5 and ERF6 play redundant roles as positive regulators of JA/Et-mediated defense against *Botrytis cinerea* in *Arabidopsis*. *PLoS One*. 7, e35995.

Robert, H.S., Grunewald, W., Sauer, M., Cannoot, B., Soriano, M., Swarup, R., et al. (2015). Plant embryogenesis requires AUX/LAX-mediated auxin influx. *Development.* 142, 702-711.

Schaller, F., Biesgen, C., Müssig, C., Altmann, T., and Weiler, E. W. (2000). 12-Oxophytodienoate reductase 3 (OPR3) is the isoenzyme involved in jasmonate biosynthesis. *Planta.* 210, 979-984.

Seo, E., Lee, H., Jeon, J., Park, H., Kim, J., Noh, Y.S., et al. (2009). Crosstalk between cold response and flowering in *Arabidopsis* is mediated through the flowering-time gene SOC1 and its upstream negative regulator FLC. *Plant Cell.* 21, 3185-3197.

Tsuchisaka, A., Yu, G., Jin, H., Alonso, J. M., Ecke,r J. R., Zhang, X., et al. (2009). A combinatorial interplay among the 1-aminocyclopropane-1-carboxylate isoforms regulates ethylene biosynthesis in *Arabidopsis thaliana*. *Genetics*. 183, 979-1003.

Turk, E. M., Fujioka, S., Seto, H., Shimada, Y., Takatsuto, S., Yoshida, S., et al. (2005). BAS1 and SOB7 act redundantly to modulate *Arabidopsis* photomorphogenesis via unique brassinosteroid inactivation mechanisms. *Plant J*. 42, 23-34

Varbanova, M., Yamaguchi, S., Yang, Y., McKelvey, K., Hanada, A., Borochov, R., et al. (2007). Methylation of gibberellins by *Arabidopsis* GAMT1 and GAMT2. *Plant Cell.* 19, 32-45.

Wang, Y., Chen, Z. H., Zhang, B., Hills, A., and Blatt, M. R. (2013). PYR/PYL/RCAR abscisic acid receptors regulate K^+ and Cl^- channels through reactive oxygen species-mediated activation of Ca^{2+} channels at the plasma membrane of intact *Arabidopsis* guard cells. *Plant Physiol.* 163, 566-577.

Wasternack, C., Goetz, S., Hellwege, A., Forner, S., Strnad, M., and Hause, B. (2012). Another JA/COI1-independent role of OPDA detected in tomato embryo development. *Plant Signal Behav.* 7, 1349-1353.

Wu, W., Wu, Y., Gao, Y., Li, M., Yin, H., Lv, M., et al. (2015). Somatic embryogenesis receptor-like kinase 5 in the ecotype Landsbergerecta of *Arabidopsis* is a functional RD LRR-RLK in regulating brassinosteroid signaling and cell death control. *Front. Plant Sci.* 6, 852.

Ye, H., Li, L., Guo, H., and Yin, Y. (2012). MYBL2 is a substrate of GSK3-like kinase BIN2 and acts as a corepressor of BES1 in brassinosteroid signaling pathway in *Arabidopsis*. *Proc. Natl. Acad. Sci.* USA. 109, 20142-20147.

Zenser, N., Ellsmore, A., Leasure, C., and Callis, J. (2001). Auxin modulates the degradation rate of Aux/IAA proteins. *Proc. Natl. Acad. Sci.* USA. 98, 11795-17800.
